# Mind the First Step: The Intrapersonal Effects of Affect on the Decision to Initiate Negotiations under Bargaining Power Asymmetry

**DOI:** 10.3389/fpsyg.2017.01313

**Published:** 2017-08-02

**Authors:** Ilias Kapoutsis, Roger Volkema, Antonia Lampaki

**Affiliations:** ^1^Department of Business Administration, School of Business, Athens University of Economics and Business Athens, Greece; ^2^Department of Management, Kogod School of Business, American University, Washington DC, United States

**Keywords:** negotiation initiation, trait affect, state affect, bargaining power, happiness, sadness, Appraisal Tendency Framework, dual-emotion situation

## Abstract

We undertook two vignette studies to examine the role of affect (trait and state) and bargaining power on initiating negotiations, an often overlooked stage of the negotiation process. Using a job negotiation opportunity, we examine three distinct phases of the initiation process—engaging a counterpart, making a request, and optimizing a request. Study 1 examines the effects of two affect dispositions (happiness and sadness), under power asymmetry (low vs. high bargaining power), on the three initiation behaviors. We found that power is pivotal to the decision to engage, request, and optimize. Also, sadness reduces the likelihood of initiation when power is high but is immaterial when power is low. In contrast, individuals who tend to be happy can reverse the adverse effect of powerlessness on requesting, but not on engaging and optimizing. However, happiness does not carry over a positive effect on negotiation initiation, over and above that of power. Study 2 investigated the role of trait affect when individuals are in power asymmetry and when they are induced with sadness or happiness. We found that those with a happy disposition initiate more (engage, request, and optimize) when power is high and experience incidental sadness. Overall, these findings qualify previous research on negotiation initiation and highlight the importance of trait affect and its interaction with state affect as additional driving forces and of power as a boundary condition.

“*for the error occurs at the beginning, and the beginning as the proverb says is half of the whole, so that even a small mistake at the beginning stands in the same ratio to mistakes at the other stages.*”

*(trans.* *Aristotle, 1944**, 1303b)*

## Introduction

Negotiation is a conflict resolution mechanism in which at least two parties decide to act to reduce perceived goal discrepancies. However, most negotiation research considers the decision to enter the negotiation as implicit and emphasizes on what happens during the negotiation and on its outcomes (Reif and Brodbeck, 2014). This perspective takes no account that a pre-negotiation phase exists in which one or more parties consider whether the situation is ripe to initiate a negotiation and communicate their preferences to their counterpart(s) (Zartman, 1989). But, why do some individuals take the first step and effectively initiate negotiations to achieve their goals, while others decide to avoid an encounter, or choose not to verbalize their request, or prefer to sub-optimize their request? As a great many decisions have an interpersonal component that requires the cooperation of other persons to accomplish, the answer to this question has direct and important implications for personal and professional success.

Despite the fact that researchers and scholars have long recognized the critical role played by early stages in many social processes (e.g., planning, problem-solving, group/team development; Tuckman, 1965; Bruner and Pomazal, 1988), the initiation phase of negotiation was for many years overlooked in models of the negotiation process, not to mention in research studies (Reif and Brodbeck, 2014, 2017). Eventually, interest in the role of gender in negotiations led some researchers to postulate differences in men’s and women’s attitudes toward negotiation, particularly as relates to making a demand or request (Babcock and Laschever, 2003; Baron, 2003; Amanatullah and Morris, 2010). Other demographic/personality factors, such as risk propensity and Machiavellianism, have since been linked to differences in propensity to initiate a negotiation (Kapoutsis et al., 2013).

In a review of research on negotiation, however, Bazerman et al. (2000) concluded that although “individual differences do influence negotiated outcomes, slight changes in situational features swamp these effects” (p. 281). Among the situational factors that could influence the initiation process are an individual’s affective state (Elfenbein, 2007; Van Kleef and Sinaceur, 2013; Reif and Brodbeck, 2014; Lerner et al., 2015; George and Dane, 2016) and the relative bargaining power of the initiator (Overbeck and Kim, 2013). These factors can differentially influence an individual’s decision to engage a counterpart, whether or not to make a request, and the nature of the request (e.g., goal optimization or sub-optimization, in the hopes of improving the chances of gaining a counterpart’s cooperation; Volkema, 2009; Kapoutsis et al., 2013). These three phases of the initiation process—engaging, requesting, and optimizing—must be studied individually as well as collectively to best understand the predictors of initiation behavior.

This research draws from the Appraisal Tendency Framework (ATF; Lerner and Keltner, 2000, 2001), which posits that emotions influence the way individuals appraise their environment toward the accomplishment of specific goals (Smith and Ellsworth, 1985; Lerner et al., 2015). Specifically, we are interested in the intrapersonal effects (i.e., when one is affected by experiencing his or her own emotions) of two discrete emotions (i.e., happiness and sadness) on the decision to initiate negotiations under bargaining power asymmetry. Our first objective is to investigate the impact of these two affect dispositions, which reflect emotions with different appraisal tendencies (happiness: a highly pleasant state involving little effort, a high level of certainty about the situation, a strong desire to pay attention, and a moderately high sense of personal control; sadness: a highly unpleasant state which is controlled by situational circumstances which are beyond individual control and associated with a high degree of anticipated effort), on negotiation initiation when bargaining power is low and high. The second is to examine the trait × state affect interaction under bargaining power asymmetry and see how their interplay (we call this the “dual-emotion situation”) may influence the decision to engage a counterpart, request and optimize.

To meet these objectives, we designed two scenario studies. Using a salary negotiation as context, Study 1 examines the impact of trait affect (happiness and sadness) and bargaining power on individuals’ decision to initiate a negotiation. Study 2 uses a similar methodology to test the interaction between state and trait affect (happiness vs. sadness) at high and low levels of relative bargaining power on each stage of the initiation process.

This research aims to advance our knowledge on negotiations in three meaningful ways. First, we offer another explanatory mechanism of why individuals decide to engage negotiations and make optimal or suboptimal requests. Although we know from prior studies that bargaining power is pivotal to negotiation initiation and needs to be accounted for (Magee et al., 2007; Kapoutsis et al., 2013; Volkema et al., 2013, 2016), there is amassing empirical evidence that demonstrates that emotions spill over their effects to decision making influencing action tendencies (for a recent review, see Lerner et al., 2015). Thus, the knowledge of how emotions impact negotiators’ decision to initiate will enhance our understanding of negotiators’ intentions in high vs. low bargaining power situations and hence indicate new practical ways to intervene or bias the decision making of counterparts.

Second, we contribute to the literature of emotions in the context of negotiations. Prior research on negotiations has focused on the interpersonal effects of emotions (e.g., Van Kleef, 2009; Van Kleef et al., 2010). Such attention is justified since emotions that counterparts display may carry social information that communicates important messages to another party and thus alternating their negotiation behavior (Van Kleef, 2016). However, emotions may as well implicitly influence cognition and hence the decisions to seize a negotiable opportunity or let it pass. With this research, we hope to provide insight into the role of two dispositional emotions (happiness and sadness) in the pre-negotiation stage.

Finally, this is one of the few studies (e.g., Winterich et al., 2010; Pe and Kuppens, 2012) to test patterns of augmentation and blunting between two quite diverse emotions, happiness and sadness, and the only one, to our knowledge, to investigate the interactive effects of the same trait and state emotions on negotiations. Literature suggests that there is a positive relationship between dispositional and state emotions (Lerner and Keltner, 2000). However, although predisposed to certain emotions, at the time of the negotiation individuals may experience congruent or incongruent incidental or integral emotions (dual emotion situation). Such a hypothesis makes a unique contribution to the negotiation literature since it shows what happens to initiation behavior, under bargaining power asymmetry, when dispositional happiness (sadness) is congruent or incongruent with state happiness (sadness). This investigation intends to open up the discussion about the dynamic interplay between state and trait affect on judgment and decision making.

## Negotiation Initiation Under Bargaining Power Asymmetry

According to the Approach-Inhibition Theory of Power, an individual’s behavioral approach system is activated when the individual has a relative power advantage, leading to a greater awareness of potential rewards and goal-directed motor behavior (Keltner et al., 2003). Besides, individuals with power may exhibit lower sensitivity to social norms and standards (Galinsky et al., 2003). Thus, people who have more relative power may well believe that their goals supersede conventional social norms and that alternatives exist if a prospective counterpart responds otherwise. An increase in goal-directed motor behavior along with a concomitant decrease in one’s sensitivity to social strictures, therefore, suggests a willingness to engage other parties who might aid in goal achievement.

While engaging can occur without requesting (as when a negotiator introduces him/herself into the conversation in the hopes that the context will spur the other party to raise a desired issue or wait until the timing is more appropriate), there is also support for a relationship between power and the second phase of the initiation process—requesting. When individuals have perceived power, they are likely to express their opinions more in social situations (Aries et al., 1983; Anderson and Berdahl, 2002). In contrast, individuals who lack power may prefer reticence with respect to goal pursuit, for fear that a request not only might be negatively received but prompt punitive action (De Dreu et al., 1998).

The third phase of initiation—optimization—also can be affected by relative power. The perception of a power advantage can imply that one’s counterpart has few if any alternatives to a negotiated agreement (e.g., no other suppliers), and therefore it is possible to optimize one’s request. This optimization, for example, can take the form of an exaggerated if not hyper-exaggerated first offer. In contrast, an initiator who him/herself lacks viable alternatives may be more inclined to lower his or her expectations and demands (Pinkley et al., 1994). In addition, prior research has shown that possessing more power increase individuals’ propensity to make a first offer and improve negotiators’ outcomes (Galinsky and Mussweiler, 2001; Magee et al., 2007).

## The Role of Emotions in Shaping Decisions: An Appraisal-Tendency Framework Perspective

Affect is a catch-all term that captures a broad range of ephemeral or enduring preferences, evaluations, moods, or emotions (Fiske and Taylor, 1991). Dispositional or trait affect refers to more stable tendencies to experience certain moods more frequently or to display specific emotional reactions to some stimulus (Scherer, 2005). In contrast, state affect reflects transitory emotional states caused by specific events that may be relevant or irrelevant to the negotiation (Barry et al., 2004; Lerner et al., 2015). While state and dispositional affect originate from different sources (situations vs. biological processes or genetic influences), research has shown that they may produce similar effects to a wide range of intrapersonal (e.g., decision making), interpersonal (e.g., performance appraisals), and organizational (e.g., cooperation, prosocial behavior) processes (Baron, 2008; Druckman and Olekalns, 2008).

Prior literature suggests that integral and incidental emotions can influence judgment and decision making (Lerner and Keltner, 2000). The former focus on the impact of emotions that are relevant to the particular situation. For example, the anticipated happiness after receiving a large bonus for the contribution to the company’s annual profits while preparing to meet with a client. The latter, which is the concern of this paper, document the influences of subjective emotional experiences that are irrelevant to the situation. For example, the anticipated sadness after seeing a fatal car accident while driving the car to an interview with an employer. Although integral emotions have shown that they can bias otherwise prudent course of action (Loewenstein et al., 2001), research on incidental emotions indicate that they can also spill over from one situation to the next (e.g., Loewenstein and Lerner, 2001; Lerner et al., 2015).

In the negotiation literature, most studies are concerned with integral affect since they have shown to convey more strategic information and thus have a stronger influence on the recipients’ judgments and behavior (Van Kleef, 2016; Hillebrandt and Barclay, 2017). Recent studies have shown that incidental emotions have stronger intrapersonal carry over than integral emotions (Schwarz and Clore, 1983; Lerner et al., 2015; Van Kleef, 2016). Nonetheless, not all emotions have a similar impact on decision making and specifically on the decision to initiate negotiations.

Research has proposed several models of emotional appraisals such as Russell’s (1980) circumplex, Frijda et al. (1989) action readiness, and Lazarus’s (1991) goal-relevance and goal congruence framework. However, the ATF (Lerner and Keltner, 2000, 2001) has moved research one step forward by suggesting that each emotion relates to a unique core appraisal that activates a cognitive predisposition (i.e., appraisal tendency). This tendency, in turn, drives individuals to appraise particular events in ways that are consistent with the emotional appraisal (Han et al., 2007). Hence, even emotions of the same valence (e.g., sadness and anger) or activation (e.g., happiness and anger) may have different influences on judgment and decision making.

To form this appraisal tendency, the ATF relies on the empirical work of Smith and Ellsworth (1985) and distinguishes emotions based on six different characteristics (Lerner et al., 2015): attentional activity, certainty about what happened, pleasantness, control, anticipated effort, and accountability. Happiness is a positive emotion associated with an elevated sense of certainty, individual control, and accountability. Moreover, those who are happy may display more attentional activity to a task, and their activation seems easier as they require less effort to overcome obstacles attached to a challenge. In contrast, sadness is a negative emotion characterized mostly by the absence of personal control, which enhances the tendency to perceive the situational elements of the prospective negotiation as responsible for its outcome (Winterich et al., 2010; Lerner et al., 2015). Also, sadness may be experienced as a burden, so individuals need to exert more effort to achieve their goal. Furthermore, sadness is associated with low attentional control which suggests that people facing a negotiation will be less concentrated to the task or repelled by it. Therefore, happiness and sadness are two discrete emotions with different underlying characteristics that each may affect differently the decision to initiate negotiations.

### Dispositional Happiness and Sadness on Negotiation Initiation under Power Asymmetry

Entering a negotiation is a decision that requires deliberation. In their review, Lerner and her colleagues (2015) suggest that emotions shape decisions through the content and depth of thought. As regards the former, the decision to initiate negotiation is typically associated with the risk of endangering valuable resources. For example, making requests to an employer may increase the perceived risk of being rejected. Indeed, how negotiators perceive risk in a given negotiation may be critical to the way they act. Prior studies have shown that action tendencies relate to risk perceptions (Lerner and Keltner, 2001; Keller et al., 2012). As for the depth of thought, the literature suggests that negative appraisals, such as sadness, may signal threat and lead to a more systematic processing as opposed to positive emotions which may signal safety and more heuristic processing (Lerner et al., 2015). However, Tiedens and Linton (2001) argued that the certainty dimension of appraisal tendencies associated with happiness rather than sadness increase heuristic processing and hence decrease attention to argument quality.

In this respect, happiness is associated with certainty and personal control which relate to risk-taking tendencies (Lerner and Keltner, 2001). Thus, we expect that under a bargaining power advantage, happy individuals will display an increased likelihood, over and above the one triggered by the positive power imbalance, of not only engaging a counterpart but also making and maximizing their requests. But, even under a relative power disadvantage, happy individuals may initiate more often as they may bias their judgment and perceive the situation as being under their control and take the risk to act to turn things around. Such behavioral intention of happy individuals may relate to the heuristic processing associated with this disposition (Tiedens and Linton, 2001). However, we doubt that their risk taking will be so intense to increase the likelihood of optimization.

Antithetically, sadness is unpleasant, it is associated with lack of personal control over the situation (such as when facing a negotiable opportunity) and requires greater effort to surmount obstacles to achieve goals. Hence, sad rather than happy individuals may become pessimistic about the situation, driving them to assess the probability of success as low (Smith and Lazarus, 1993). Therefore, we expect that this pessimistic approach will prevent them from activating and taking risks especially when relative power is low. In this case, the likelihood of engaging, requesting, and optimizing would further decrease. Also, even when power asymmetry is positive, their dispositional sadness may attenuate power’s positive impact on negotiation initiation intentions. Nonetheless, we expect that this buffering effect will be most apparent in optimizing and requesting and not in engaging. The reason for this differentiation is that engaging under a relative power advantage seems a risk-free choice even for sad individuals.

## The Dual-Emotion Situation and its Impact on Negotiation Initiation Under Bargaining Power Asymmetry

The previous discussion made clear that trait affect may influence the decision to initiate negotiations. Surprisingly, prior research on decision making has focused on the action tendencies of either dispositional affect or state affect (as general tendencies or discrete emotions). Yet, trait and state affect may interact within the same individual. For example, a job candidate may have a happy disposition, but be in a sad mood during an interview because of a previous integral or incidental event. This interplay of trait and state affect raises new questions about the implications of their interaction in decision making and negotiation initiation, in particular.

The tenet that emotional experiences may influence each other across time is not new, although empirical evidence are still sparse (e.g., Branscombe, 1985; Neumann et al., 2001; Winterich et al., 2010; Pe and Kuppens, 2012). For example, Branscombe (1985) relied on the incompatible response model (Baron, 1976, 1984) and argued that when one emotion immediately follows another of the same valence, these will sum and result in a more intense response to the second stimulus. In another study, Winterich et al. (2010) argued that since appraisal tendencies influence judgment and cognition, they may as well influence an existing appraisal. In their study, they focused on contrasting appraisals that inhibit previous emotions, which they referred to as emotional blunting. Specifically, they used sadness and anger, two emotions negatively valenced that mainly differ in situational control (i.e., sadness is characterized by appraisals of situational control whereas anger by appraisals of individual control) and found that sadness will blunt the elicitation of sadness in a subsequent event that should elicit anger and in turn carry over to optimism in risk estimates. In addition, Pe and Kuppens (2012) suggested that one emotion can either augment or blunt the experience of another subsequent emotion. In their study, they found that emotions of similar valence augmented the experience of another whereas they blunted it in the case of emotions of the opposite valence.

Based on the previous discussion, we expect that when state happiness (sadness) is congruent with dispositional happiness (sadness), the effect of the latter will be amplified. Thus, being in a happy (sad) state, while having a happy (sad) disposition, will increase (decrease) the likelihood of initiating, especially when bargaining power is also high (low). In contrast, we expect that when state happiness (sadness) is infused to a sad (happy) individual, the negative (positive) effect of dispositional sadness (happiness) on negotiation initiation will attenuate, even when bargaining power is low (high).

## The Present Research

To test these propositions, we designed two scenario studies. The first tests the impact of dispositional affect (happiness and sadness) on three distinct stages of negotiation initiation (engage, request, optimize). In the second study, we manipulate state affect (happiness vs. sadness) and test its interplay with dispositional affect (happiness and sadness) on each stage of the negotiation initiation process. In both studies, bargaining power is manipulated to test these effects under power asymmetry (high vs. low relative bargaining power). To our knowledge, this research is the first to investigate the role of affect on negotiation initiation and one of the few that empirically tests the interaction between trait and state affect (e.g., Van Knippenberg et al., 2010). Finally, the current research is unique since the role of affect is tested in light of power asymmetries.

### Ethics Statement

An ethics approval was not needed for this type of study according to institutional and national guidelines. In our cover letter to participants, we explicitly stated that participation was voluntary and that anonymity was ensured as no identifying, personal, or health related information were collected. Furthermore, all participants were informed that they had the option to withdraw from the survey at any moment while at the beginning of the survey they indicated their consent by checking a relevant checkbox.

### Study 1

#### Participants and Procedure

The participants were 108 full-time employees. Fifty-two percent were females, with a mean age of 34.98 years (*SD*_age_ = 12.2) and 2.37 years of work experience (*SD*_tenure_ = 2.06). They were recruited through Prolific Academic, an online labor marketplace in which employers can recruit workers to complete short tasks for a small fee. Recent studies have shown that such crowdsourcing online marketplaces (e.g., Amazon’s MTurk, Prolific Academic, Crowdflower) for recruitment of subjects in research are a reliable and cost-efficient method of getting high-quality data associated with significant benefits (Rand et al., 2012). The most prominent benefits are that the demographic characteristics of their workers are more representative of non-college populations and that such platforms allow other researchers to replicate findings (Buhrmester et al., 2011; Rand et al., 2012). Based on recent evidence (Peer et al., 2017), the Prolific Academic platform managed to reproduce known effects of all the tested tasks, its workers exhibited lower propensity to engage in dishonest behaviors, while the data reliability (i.e., passing attention checks) was high.

For this study, we chose few pre-screening requirements. Participants’ pool consisted of English speaking Caucasians, who worked full-time. To be eligible to participate, they also had to display at least 80% approval rate in prior tasks (an indication of the quality of their responses) and had at least two successful submissions. The total number of eligible participants at the time of the data collection was 11,420. To compensate for their time, we offered a baseline payment of £1 (£10/hour), which was well above the lower (£5/hour) or the suggested £6.5/7.5 hourly rate. The average time needed to complete the survey was 6 min.

To ensure that all participants would display a satisfactory level of attention and hence reduce noise in the data, we included two attention and four recall checks scattered into different sections of the online survey. For example, after reading the scenario participants had to respond to four questions to verify that they recall the information presented in the scenario (e.g., “*Is this job your top choice?*,” “*Do you expect any other offer from other companies?*”) or, after providing the demographic information, they had to choose the value that equals 3 × 2. Participants who failed to pass one attention or recall check (i.e., 30.3%) were excluded from the study after being debriefed of the reason. The probability of passing all attentions checks by providing automated answers was practically 0 (i.e., below 0.1%).

In the first part of the survey, participants were asked to complete the measures of happiness and sadness with other measures and demographic characteristics to avoid signaling the actual purpose of the study and reduce attribution bias. Then, we randomly presented a vignette describing either a low (*N* = 54) or high (*N* = 54) bargaining power situation and instructed them to take some time to think of the situation and let themselves react as if this was actually happening to them. After reading the scenario and passing the necessary attention and recall checks, respondents ranked four alternatives that reflected different stages of negotiation initiation (avoid engaging, engaging, requesting, optimizing).

##### Assessing dispositional affect

Dispositional affect encompasses many different positive and negative emotions. In this study, we focused on two primary and relevant to negotiations emotions, happiness and sadness. To measure perceived happiness and perceived sadness, we used the joviality (eight items) and sadness (five items) dimensions from the Positive Affect Negative Affect Scale (PANAS; Watson et al., 1988). On a 5-point scale, ranging from “very slightly or not at all” (1) to “extremely” (5), participants assessed how they felt during the past few weeks by responding to the words “happy,” “joyful,” “delighted,” “cheerful,” “excited,” “enthusiastic,” “lively,” and “energetic,” which assess happiness (joviality dimension of PANAS), and to the words “sad,” “blue,” “downhearted,” “alone,” and “lonely,” which measure sadness. Reliability estimates for both measures were adequate (happiness: α = 0.93; sadness: α = 0.91).

##### Manipulation of bargaining power

Participants were asked to read a scenario describing a salary negotiation (see **Appendix**). To manipulate bargaining power, we altered the scenario in the following aspects: the desire for the position, the existence of alternative offers, and the existence of other competent candidates. For the low power condition, the scenario mentioned that the offered job was one of their top choices, while no other offer was on the table, and other competent candidates were competing for the position. In contrast, the high bargaining power condition mentioned that the job was respectable but not among their first choices, they had other offers pending, and they were the most competent candidates for the position.

##### Assessing negotiation initiation

We instructed participants to put themselves in the described position and react as if this was actually happening to them. Then we prompted them to choose their most preferred response among four behavioral alternatives (presented in randomized order) that described different levels of initiation behavior (see **Appendix**): (a) no engagement of a counterpart, (b) engagement without making a request, (c) engagement with a suboptimal request, and (d) engagement with an optimized request. Then, based solely on their preferred alternative, each participant was scored for engaging (0 or 1), requesting (0 or 1), and optimizing (0 or 1). This way, individuals who chose to optimize as their preferred response, received “1” for engaging, “1” for requesting, and “1” for optimizing, while those who preferred to engage received “1” for engaging and “0” for the other two initiation behaviors.

##### Controls

Prior studies suggest that males are less likely to initiate a negotiation (e.g., Babcock et al., 2006). Also, older participants have been negatively associated with the propensity to initiate negotiations (Volkema and Fleck, 2012). Therefore, we included gender and age as covariates in our study.

#### Treatment of the Data

To test the impact of dispositional affect and bargaining power on the three binary dependent variables we employed hierarchical logistic regression. Controls and main effects were entered in Step 1 and the interaction terms were included in Step 2. All continuous variables were standardized before creating the interaction terms to avoid multicollinearity with their main effects (Aiken and West, 1991) and enhance the interpretability of the findings.

#### Results

##### Manipulation checks

To check whether we manipulated bargaining power correctly we asked respondents to indicate their perceived bargaining power on a visual scale from 0 (the company has all the power) to 10 (I have all the power) after they had read the scenario. Participants assigned to the low power condition reported a significantly lower value of bargaining power compared to those in the high power condition (*M*_high power_ = 7.65, *SD*_high power_ = 1.53; *M*_low power_ = 4.09, *SD*_low power_ = 1.90; *t*(106) = 10.72, *p* < 0.001).

**Table 1** presents the descriptive statistics and bivariate correlations between the study variables. In general, 68.5% of the participants chose to engage a counterpart, 44.4% to make a suboptimal request, and 27.8% to optimize their request. **Tables 2**–**4** exhibits the logistic regression results.

**Table 1:** Means, standard deviation, and correlation between variables (Studies 1 and 2).

	Mean	*SD*	1	2	3	4	5	6	7	8
1. Sex	1.52 1.47	0.50 0.50								
2. Age	34.98 35.29	12.20 12.34	0.19^†^ 0.31							
3. Bargaining power	0.50 0.51	0.50 0.50	0.15 0.04	0.12 –0.17^∗^						
4. Perceived happiness	2.64 2.60	0.89 0.87	–0.02 –0.06	–0.09 0.04	–0.00 –0.11					
5. Perceived sadness	2.28 2.41	1.05 0.99	–0.02 0.16^∗^	–0.12 –0.20^∗∗^	–0.09 0.17^∗^	–0.46^∗∗^ –0.55^∗∗^				
6. State affect	– 0.49	– 0.50	– –0.05	– –0.08	– –0.01	– 0.02	– –0.01			
7. Engaging	0.69 0.77	0.47 0.42	–0.06 –0.13^†^	0.10 –0.03	0.24^∗^ 0.31^∗∗^	0.07 0.16^∗^	–0.19^∗^ –0.10	– 0.01		
8. Requesting	0.44 0.41	0.50 0.49	–0.07 –0.11	0.14 0.01	0.34^∗∗^ 0.16^∗^	0.10 0.20^∗^	–0.18^†^ –0.22^∗∗^	–0.02	0.61^∗∗^ 0.46^∗∗^	
9. Optimizing	0.28 0.21	0.45 0.41	–0.02 –0.06	0.15 0.13^†^	0.37^∗∗^ 0.19^∗^	0.16^†^ 0.21^∗∗^	–0.40^∗∗^ –0.22^∗∗^	–0.05	0.42^∗∗∗^ 0.29^∗∗^	0.69^∗∗^ 0.63^∗∗^

*Study 1: *N*_*sample 1*_ = 108 (first row); Study 2: *N*_*sample 2*_ = 168 (second row). Sex: 0 = male, 1 = female; bargaining power: 0 = low, 1 = high; state affect: 0 = sad, 1 = happy; engaging/requesting/optimizing: 0 = no, 1 = yes. ^∗∗∗^*p* < 0.001, ^∗∗^*p* < 0.01, ^∗^*p* < 0.05, ^†^*p* < 0.10.*

**Table 2:** Binary logistic regression results for engaging (Study 1).

	Engage (*N* = 108)			Low bargaining power (*N* = 54)			High bargaining power (*N* = 54)		
Variables	β (*SE*)	Wald	OR	β (*SE*)	Wald	OR	β (*SE*)	Wald	OR
**Model 1^a^**									
Constant	0.33 (0.29)	1.36	1.40	0.31 (0.28)	1.22	1.37	1.67 (0.43)	15.39^∗∗∗^	5.32
Sex	–0.26 (0.23)	1.26	0.77	–0.35 (0.30)	1.33	0.71	–0.14 (0.41)	0.13	0.87
Age	0.21 (0.24)	0.74	1.23	0.31 (0.32)	0.98	1.37	0.16 (0.43)	0.14	1.17
Bargaining power (P)	1.09 (0.45)	5.71^∗∗∗^	2.96						
Happiness (H)	0.00 (0.25)	0.00	1.00	0.27 (0.34)	0.61	1.30	–0.34 (0.46)	0.55	0.71
Sadness (S)	–0.38 (0.25)	2.29	0.69	0.27 (0.33)	0.68	1.31	–1.17 (0.44)	7.08^∗∗^	0.31
Model fit (H–L) χ^2^ (*df*)	10.54(8), *p* = 0.23			6.70(8), *p* = 0.57			9.79(8), *p* = 0.28		
*R*^2^ (Cox & Snell)	0.10			0.04			0.17		
*R*^2^ (Nagelkerke)	0.14			0.06			0.26		
Model accuracy	0.71			0.63			0.82		
**Model 2^b^**									
P × H	–0.56 (0.58)	0.93	0.57						
P × S	–1.35 (0.53)	6.44^∗^	0.26						
Model fit (H–L) χ^2^	8.09(8), *p* = 0.42								
Δ*R*^2^ (Cox & Snell)	0.06								
Δ*R*^2^ (Nagelkerke)	0.08								
Δmodel accuracy	0.02								

*^a^Statistics based on step 1 only; ^b^statistics based on step 2 only. β, beta coefficient (in log-odds units); SE, standard error; Wald, Wald chi-square; OR, odds ratio; H–L = Hosmer–Lemeshow goodness of fit. ^∗∗∗^*p* < 0.001, ^∗∗^*p* < 0.01, ^∗^*p* < 0.05.*

**Table 3:** Binary logistic regression results for requesting (Study 1).

	Request (*N* = 108)			Low bargaining power (*N* = 54)			High bargaining power (*N* = 54)		
Variables	β (*SE*)	Wald	OR	β (*SE*)	Wald	OR	β (*SE*)	Wald	OR
**Model 1^a^**									
Constant	–1.01 (0.32)	10.04^∗∗^	0.36	–1.10 (0.34)	0.05^∗∗^	0.33	0.52 (0.30)	2.87	1.67
ex	–0.35 (0.23)	2.46	0.70	–0.51 (0.36)	2.00	0.60	–0.09 (0.31)	0.09	0.91
Age	0.29 (0.23)	1.64	1.34	0.23 (0.37)	0.38	1.26	0.51 (0.33)	2.48	1.67
Bargaining power (P)	1.49 (0.44)	11.52^∗∗^	4.45						
Happiness (H)	0.15 (0.24)	0.38	1.16	0.72 (0.40)	3.29^†^	2.06	–0.55 (0.36)	2.34	0.58
Sadness (S)	–0.25 (0.24)	1.07	0.78	0.15 (0.40)	0.15	1.16	–0.63 (0.36)	3.12^†^	0.53
Model fit (H–L) χ^2^ (*df*)	4.98(8), *p* = 0.76			7.54(8), *p* = 0.48			10.69(8), *p* = 0.22		
*R*^2^ (Cox & Snell)	0.16			0.12			0.13		
*R*^2^ (Nagelkerke)	0.21			0.17			0.18		
Model accuracy	0.67			0.69			0.61		
**Model 2^b^**									
P × H	–1.30 (0.53)	5.97^∗^	0.27						
P × S	–0.80 (0.53)	2.24	0.45						
Model fit (H–L) χ^2^	8.78(8), *p* = 0.36								
Δ*R*^2^ (Cox & Snell)	0.05								
Δ*R*^2^ (Nagelkerke)	0.07								
Δmodel accuracy	0.00								

*^a^Statistics based on step 1 only; ^b^statistics based on step 2 only. β, beta coefficient (in log-odds units); SE, standard error; Wald, Wald chi-square; OR, odds ratio; H–L, Hosmer–Lemeshow goodness of fit. ^∗∗^*p* < 0.01, ^∗^*p* < 0.05, ^†^*p* < 0.10.*

**Table 4:** Binary logistic regression results for optimizing (Study 1).

	Optimize (*N* = 108)			Low bargaining power (*N* = 54)			High bargaining power (*N* = 54)		
Variables	β (*SE*)	Wald	OR	β (*SE*)	Wald	OR	β (*SE*)	Wald	OR
**Model 1^a^**									
Constant	–2.49 (0.51)	3.35^∗∗∗^	0.08	–2.31 (0.52)	19.52^∗∗∗^	0.10	–0.63 (0.41)	2.32	0.53
Sex	–0.39 (0.28)	1.92	0.68	–0.20 (0.50)	0.16	0.82	–0.55 (0.38)	2.09	0.58
Age	0.21 (0.26)	0.65	1.24	0.25 (0.53)	0.22	1.29	0.31 (0.34)	0.81	1.36
Bargaining power (P)	2.00 (0.58)	11.74^∗∗^	7.41						
Happiness (H)	0.02 (0.30)	0.01	1.02	0.73 (0.55)	1.74	2.08	–0.46 (0.40)	1.35	0.63
Sadness (S)	–1.22 (0.39)	10.01^∗∗^	0.29	–0.07 (0.59)	0.02	0.93	–2.17 (0.68)	10.22^∗∗^	0.11
Model fit (H–L) χ^2^ (*df*)	8.04(8), *p* = 0.43			11.28(8), *p* = 0.19			9.58(8), *p* = 0.30		
*R*^2^ (Cox & Snell)	0.28			0.06			0.35		
*R*^2^ (Nagelkerke)	0.41			0.11			0.47		
Model accuracy	0.81			0.89			0.70		
**Model 2^b^**									
P × H	–1.16 (0.65)	3.17^†^	0.31						
P × S	–1.89 (0.88)	4.66^∗^	0.15						
Model fit (H–L) χ^2^	10.499(8), *p* = 0.23								
Δ*R*^2^ (Cox & Snell)	0.04								
Δ*R*^2^ (Nagelkerke)	0.06								
Δmodel accuracy	0.00								

*^a^Statistics based on step 1 only; ^b^statistics based on step 2 only. β, beta coefficient (in log-odds units); SE, standard error; Wald, Wald chi-square; OR, odds ratio; H–L, Hosmer–Lemeshow goodness of fit. ^∗∗∗^*p* < 0.001, ^∗∗^*p* < 0.01, ^∗^*p* < 0.05, ^†^*p* < 0.10.*

As regards the decision to engage the negotiation (**Table 2**), we found a significant effect for bargaining power. Odds ratio (OR) value, 2.96, *p* = 0.02, 95% CI (1.22, 7.21), indicates that the more relative power participants have, the more likely (2.96 times) they are to engage a negotiation. More specifically, those low on power had a probability of 58.18% to engage a negotiation, whereas those high on power had an 80.53% chance. Although all other main effects were insignificant, the interaction of bargaining power × sadness was significant. To probe this interaction, we ran two binary logistic regressions—one for low and one for high bargaining power—in which our two dispositional emotions were regressed on engaging along with the other covariates. Our analysis showed that sadness was the only significant predictor when power was high, OR = 0.31, *p* = 0.01, 95% CI (0.13, 0.74), such that sad participants who possess more relative bargaining power are 3.23 less likely to engage. In fact, for one unit increase in sadness, the probability of engaging for powerful participant drops to 62.25%.

Likewise, for requesting (**Table 3**), bargaining power is a significant predictor, OR = 4.45, *p* < 0.01, 95% (1.879, 10.548), such that those who possess it are 4.45 times for likely to make a suboptimal request (probability of requesting: 26.70% when power is low and 61.77% when power is high). At Step 2, we found a significant interaction of power × happiness. To understand the nature of this interaction, we performed two binary regressions—one for low and one for high bargaining power. The results indicate a marginally significant effect of happiness when bargaining power is low, OR = 2.06, *p* = 0.07, 95% CI (0.94, 4.49), such that happy participants are 2.06 times more likely to make a request (probability of requesting increases to 40.61% when happiness increases by one unit). Nonetheless, for those high on power, this positive effect was reversed, although it was not statistically significant. In addition, we found that sadness had a marginally significant effect, OR = 0.58, *p* = 0.08, 95% CI (0.27, 1.07). Thus, sad participants are 1.72 times less likely to make a request (probability of requesting drops to 49.18% for one unit increase in sadness).

As for optimizing (**Table 4**), we found significant main effects for bargaining power, OR = 7.41, *p* < 0.01, 95% CI (2.36, 23.29), and sadness, OR = 0.29, *p* < 0.01, 95% CI (0.14, 0.63). Such results indicate that powerful participants are 7.41 more likely to optimize their request (probability for optimizing is 7.68% when power is low and 38.11% when power is high), while sad participants are 3.40 less likely to optimize. Most interestingly, we found significant interaction effects for power × sadness. To probe this interaction, we again ran two binary logistic regressions (high vs. low bargaining power). When bargaining power is high, sadness has a negative impact on the decision to optimize the request, OR = 0.11, *p* < 0.01, 95% CI (0.03, 0.43), such that sad individuals are 9.09 times less likely to optimize their requests (probability of optimizing drops to 5.75% for one unit increase in sadness). Happiness had a negative, but insignificant effect. At low bargaining power, both dispositional emotions had an insignificant impact on requesting.

As a robustness check to our findings, we excluded all controls from the analysis as they had a minor impact on the dependent variables and rerun the logistic regressions. Their exclusion did not substantially change our results. The effects remained statistically significant and in the same pattern as reported above.

#### Discussion

Study 1 aimed at advancing our knowledge on the role of two affect dispositions (happiness and sadness), one positive emotion with high activation and one negative emotion with low activation, and bargaining power on three stages of the negotiation initiation (engaging, requesting, and optimizing). Our findings corroborate our expectations that bargaining power is pivotal to negotiation initiation. However, affect dispositions are capable of restraining the dominant role of bargaining power. Specifically, we found that increased levels of sadness lower the probability of powerful individuals to engage a counterpart, make a request, or optimize the request. More aptly put, perceptions of sadness may block activation of a negotiable opportunity despite the relative power advantage. Being sad has no downward spiral effect when relative power is low. On the contrary, the results indicate that perceived happiness can surpass the high activation threshold that low bargaining power sets, but only in the stage of requesting. As regards engaging and optimizing, no matter the level of perceived happiness, having low power seems critical. Finally, similar to the insignificant effect of sadness when power is low, happiness failed to produce an upward spiral to initiation when bargaining power is high.

### Study 2

#### Participants and Design

As in Study 1, we employed workers from Prolific Academic with the same sample characteristics and used the same pre-screening criteria plus excluding those who had participated in the previous study. This time, 168 full-time employees were recruited and provided valid responses (33.8% of those initially recruited failed to pass the attention and recall questions and were excluded from the analyses; 47% women; *M*_age_ = 35.29, *SD*_age_ = 12.34; *M*_work experience_ = 2.52, *SD*_work experience_ = 2.17). They were randomly assigned to one to four cells of a 2 (feeling happy vs. feeling sad) × 2 (high vs. low bargaining power) between-subjects design. To compensate for workers’ time, we offered a baseline payment of £1.2 (£10.30/hour) and the average time needed to complete the survey was 8 min.

The second study aimed at documenting the impact of the same dispositional emotions at two different conditions of state (incidental) affect (happiness vs. sadness) and bargaining power (low vs. high) on each stage of negotiation initiation (engage, request, and optimize). We induced state affect using hypothetical incidents describing happy or sad situations (see below) and we manipulated bargaining power as in Study 1. Also, we assessed dispositional affect (happiness and sadness) and negotiation initiation (engage, request, optimize) using the same measures as in Study 1. The Cronbach alphas for happiness (joviality dimension of PANAS) and sadness were 0.94 and 0.90, respectively.

##### Manipulation of state affect

To induce happiness or sadness, which represent two common affective states, participants read either of two scenarios developed by Larsen and Ketelaar (1991) and then took a couple of minutes to imagine the situation as vividly as possible. The positive affect condition required participants to imagine themselves winning €50,000 in a lottery and then taking a vacation to Hawaii. The negative affect condition asked participants to imagine being embarrassingly expelled from school and then having a close friend die from a painful, incurable disease. At the background of each scenario, we placed a picture that was relevant to the emotion. Then, we requested from those participants who read the happy situation to write how they would spend their time in Hawaii and to those who read the sad one to write about a similar experience that had happened to them in the past. To check the embedded manipulation and use it as a repeatable measure to assess changes in their affect throughout the experiment, we used a bipolar scale. Participants indicated how happy or how sad they felt by moving a marker of a visual analog scale right or left in one-unit intervals along a continuum from 0 (sad) to 10 (happy).

#### Treatment of the Data

Using a binary logistic regression, we first regressed bargaining power, state, and dispositional affect on each of the three binary dependent variables (initiate, request, optimize; see **Table 5**). Then, for each dependent variable (see **Tables 6**–**8**), we ran four binary logistic regressions to test the effect of dispositional affect (happiness and sadness) depending on different power conditions (high vs. low bargaining power) and state affect (feeling sad vs. feeling happy). Results in Study 1 showed that controls (i.e., gender and age) had insignificant effects on the decision to initiate negotiations. Thus, to reduce the complexity of the model and increase statistical power, we decided to exclude them from all subsequent analyses.

**Table 5:** Binary logistic regression of bargaining power, state and trait affect on negotiation initiation (engage, request, optimize; Study 2).

	Engage			Request			Optimize		
Variables	β (*SE*)	Wald	OR	β (*SE*)	Wald	OR	β (*SE*)	Wald	OR
Constant	0.48 (0.31)	2.34	1.61	–0.86 (0.30)	8.35^∗∗^	0.42	–2.04 (0.40)	26.24^∗∗∗^	0.13
Bargaining power (P)	1.88 (0.46)	17.02^∗∗∗^	6.52	0.91 (0.34)	7.11^∗∗^	2.49	1.28 (0.43)	8.82^∗∗^	3.58
Affect (A)	0.05 (0.40)	0.01	1.05	–0.02 (0.33)	0.00	0.98	–0.28 (0.40)	0.48	0.76
Happiness (H)	0.43 (0.25)	3.05^†^	1.54	0.25 (0.20)	1.61	1.29	0.34 (0.26)	1.93	1.40
Sadness (S)	–0.22 (0.24)	0.89	0.34	–0.43 (0.21)	4.42^∗∗^	0.65	–0.54 (0.26)	4.26^∗^	0.13
Model fit (H–L) χ^2^ (*df*)	12.25(8), *p* = 0.14			9.59(8), *p* = 0.30			4.448(8), *p* = 0.82		
*R*^2^ (Cox & Snell)	0.14			0.10			0.12		
*R*^2^ (Nagelkerke)	0.21			0.13			0.18		
Model accuracy	0.79			0.65			0.79		

*^a^Statistics based on step 1 only; ^b^statistics based on step 2 only. β, beta coefficient (in log-odds units); SE, standard error; Wald, Wald chi-square; OR, odds ratio; H–L, Hosmer–Lemeshow goodness of fit. ^∗∗∗^*p* < 0.001, ^∗∗^*p* < 0.01, ^∗^*p* < 0.05, ^†^*p* < 0.10.*

**Table 6:** Binary logistic regression of happiness and sadness (trait affect) on engaging at different levels of bargaining power (high vs. low) and state affect (sadness vs. happiness) (Study 2).

	Bargaining power (low) (*N* = 82)						Bargaining power (high) (*N* = 86)					
	State affect (sad) (*N* = 41)			State affect (happy) (*N* = 41)			State affect (sad) (*N* = 44)			State affect (happy) (*N* = 42)		
Variables	β (*SE*)	Wald	OR	β (*SE*)	Wald	OR	β (*SE*)	Wald	OR	β (*SE*)	Wald	OR
Constant	0.75 (0.37)	4.18^∗^	2.11	0.46 (0.32)	1.98	1.58	2.40 (0.68)	12.61^∗∗∗^	11.05	2.71 (0.68)	15.78^∗∗∗^	15.06
Happiness (H)	0.68 (0.43)	2.53	1.97	0.23 (0.37)	0.37	1.25	1.69 (0.92)	3.39^†^	5.45	–0.54 (0.66)	0.67	0.58
Sadness (S)	–0.38 (0.41)	0.87	0.68	–0.11 (0.36)	0.10	0.89	0.49 (0.76)	0.42	1.64	0.13 (0.64)	0.04	1.14
Model fit (H–L) χ^2^ (*df*)	9.93(8), *p* = 0.27			10.23(8), *p* = 0.25			8.02(8), *p* = 0.43			8.87(8), *p* = 0.35		
*R*^2^ (Cox & Snell)	0.15			0.02			0.13			0.02		
*R*^2^ (Nagelkerke)	0.21			0.03			0.23			0.05		
Model accuracy	0.73			0.56			0.86			0.93		

*β, beta coefficient (in log-odds units); SE, standard error; Wald, Wald chi-square; OR, odds ratio; H–L, Hosmer–Lemeshow goodness of fit. ^∗∗∗^*p* < 0.001, ^∗∗^*p* < 0.01, ^∗^*p* < 0.05, ^†^*p* < 0.10.*

**Table 7:** Binary logistic regression of happiness and sadness (trait affect) on requesting at different levels of bargaining power (high vs. low) and state affect (sadness vs. happiness) (Study 2).

	Bargaining power (low) (*N* = 82)						Bargaining power (high) (*N* = 86)					
	State affect (sad) (*N* = 41)			State affect (happy) (*N* = 41)			State affect (sad) (*N* = 44)			State affect (happy) (*N* = 42)		
Variables	β (*SE*)	Wald	OR	β (*SE*)	Wald	OR	β (*SE*)	Wald	OR	β (*SE*)	Wald	OR
Constant	–0.60 (0.34)	3.10^†^	0.55	–0.88 (0.34)	6.62^∗^	0.41	–0.28 (0.43)	0.42	0.76	0.10 (0.31)	0.10	1.10
Happiness (H)	0.17 (0.40)	0.19	1.19	0.02 (0.39)	0.00	0.97	2.08 (0.83)	6.23^∗^	7.97	–0.34 (0.35)	0.97	0.71
Sadness (S)	–0.49 (0.42)	1.35	0.62	–0.09 (0.39)	0.06	0.91	–0.25 (0.66)	0.14	0.78	–0.11 (0.34)	0.10	0.90
Model fit (H–L) χ^2^ (*df*)	9.32(8), *p* = 0.32			6.20(8), *p* = 0.63			5.30(8), *p* = 0.73			7.78(8), *p* = 0.46		
*R*^2^ (Cox & Snell)	0.06			0.00			0.45			0.02		
*R*^2^ (Nagelkerke)	0.09			0.00			0.61			0.03		
Model accuracy	0.66			0.71			0.82			0.55		

*β, beta coefficient (in log-odds units); SE, standard error; Wald, Wald chi-square; OR, odds ratio; H–L, Hosmer–Lemeshow goodness of fit. ^∗∗^*p* < 0.01, ^∗^*p* < 0.05, ^†^*p* < 0.10.*

**Table 8:** Binary logistic regression of happiness and sadness (trait affect) on optimizing at different levels of bargaining power (high vs. low) and state affect (sadness vs. happiness) (Study 2).

	Bargaining power (low) (*N* = 82)						Bargaining power (high) (*N* = 86)					
	State affect (sad) (*N* = 41)			State affect (happy) (*N* = 41)			State affect (sad) (*N* = 44)			State affect (happy) (*N* = 42)		
Variables	β (*SE*)	Wald	OR	β (*SE*)	Wald	OR	β (*SE*)	Wald	OR	β (*SE*)	Wald	OR
Constant	–2.16 (0.68)	9.98^∗∗^	0.12	–3.16 (1.04)	9.19^∗∗^	0.04	–1.36 (0.49)	7.69^∗∗^	0.26	–0.97 (0.36)	7.32^∗∗^	0.38
Happiness (H)	0.24 (0.57)	0.18	1.28	1.15 (0.79)	2.09	3.15	1.68 (0.73)	5.34^∗^	5.38	–0.52 (0.41)	1.58	0.59
Sadness (S)	–1.40 (0.79)	3.15^†^	0.25	–0.84 (0.93)	0.82	0.43	–0.06 (0.66)	0.01	0.94	–0.07 (0.41)	0.03	0.93
Model fit (H–L) χ^2^ (*df*)	13.53(8), *p* = 0.10			9.91(8), *p* = 0.27			6.07(8), *p* = 0.64			6.59(8), *p* = 0.58		
*R*^2^ (Cox & Snell)	0.13			0.13			0.32			0.04		
*R*^2^ (Nagelkerke)	0.22			0.28			0.45			0.06		
Model accuracy	0.83			0.90			0.82			0.71		

*β, beta coefficient (in log-odds units); SE, standard error; Wald, Wald chi-square; OR, odds ratio; H–L, Hosmer–Lemeshow goodness of fit. ^∗∗^*p* < 0.01, ^∗^*p* < 0.05, ^†^*p* < 0.10.*

#### Results

##### Manipulation checks

To assess the influence of our manipulations, we run several independent samples *t*-tests. Participants who were assigned to the low power condition reported a lower mean value, *M*_high power_ = 7.48, *SD*_high power_ = 2.10; *M*_low power_ = 5.35, *SD*_low power_ = 2.20; *t*(166) = 6.42, *p* < 0.001. To validate the manipulation of state affect (sad vs. happy), we repeatedly checked respondents’ level of affectivity using another visual analog scale (0 = feeling sad, 10 = feeling happy). The first (T0) was before introducing the scenarios to respondents, the second (T1) right after the manipulation of state affect, the third (T2) after the manipulation of bargaining power, and the last (T3) after respondents had chosen their behavioral response. As expected, at T0, respondents reported similar values of state affect, *M*_sad_ = 6.33, *SD*_sad_ = 2.14, *M*_happy_ = 6.29, *SD*_happy_ = 1.89, *t*(166) = 0.13, *p* = 0.90. In addition, we ran a one-way ANOVA to test whether participants in different bargaining and affect manipulation conditions would report similar levels of state happiness or sadness. Again, the analysis indicated that in all four cases (i.e., high power and happy, high power and sad, low power and happy, lower power and sad) participants reported similar levels of state affect at T0, *F*(3,164) = 1.44, *p* = 0.23. At T1, the results indicate that those induced with happiness reported a higher value compared to those induced with sadness, *M*_sad_ = 3.94, *SD*_sad_ = 1.91, *M*_happy_ = 7.02, *SD*_happy_ = 1.98, *t*(166) = -10.28, *p* < 0.001. At this stage, one reasonable question is whether state affect would be influenced by the manipulation of bargaining power. The *t*-test indicated that the state affect manipulation remained valid even after the manipulation of bargaining power, *M*_sad_ = 4.91, *SD*_sad_ = 1.89, *M*_happy_ = 5.86, *SD*_happy_ = 2.03, *t*(166) = -3.14, *p* < 0.01. Moreover, even after providing their behavioral responses, participants who were induced with happiness reported higher values compared to those who were induced with sadness, *M*_sad_ = 5.18, *SD*_sad_ = 1.97, *M*_happy_ = 5.78, *SD*_happy_ = 1.99, *t*(166) = -1.98, *p* < 0.05. Finally, we performed a *t*-test to compare whether those in the happy state perceived that they had more power compared to those in the sad one and we found no supporting evidence, *M*_sad_ = 6.54, *SD*_sad_ = 2.39, *M*_happy_ = 6.23, *SD*_happy_ = 2.39, *t*(166) = 0.85, *p* = 0.40. In addition to these tests, we performed some paired-samples *t*-tests to check whether the manipulation of state affect managed to induce the intended mood compared to their initial mood effectively. Thus, we compared the responses of those in the happy state between T0 and T1 and we found that their values increased after the manipulation, *M*_T0_ = 6.29, *SD*_T0_ = 1.89, *M*_T1_ = 7.02, *SD*_T1_ = 1.98, t(82) = -6.06, *p* < 0.001. Similarly, those who received the sadness induction reported lower values after the manipulation, *M*_T0_ = 6.33, *SD*_T0_ = 2.14, *M*_T1_ = 3.94, *SD*_T1_ = 1.91, *t*(84) = 9.71, *p* < 0.001. Overall, these results provide robust evidence that our manipulation of state affect (happiness vs. sadness) was successful and independent from the manipulation of bargaining power.

**Table 1** presents the descriptive statistics and bivariate correlations between study variables. Overall, 76.8% of participants chose to engage a negotiation, 41.1% to make a suboptimal request, and 21.4% to optimize it. **Table 5** exhibits the results of the traits and state affect as well as bargaining power on all three stages of the initiation process (engaging, requesting, optimizing).

##### Engage the negotiation

The binary logistic regression including affect (dispositional and state) and bargaining power as main effects showed some significant effects for power, OR = 6.52, *p* < 0.001, 95% CI (2.68, 15.90), and happiness as a trait, OR = 1.54, *p* = 0.08, 95% CI (0.95, 2.49). Specifically, powerful and happy participants are 6.52 times and 1.54 times, respectively, more likely to engage a negotiation. We found no effect for state affect.

In addition, we run four binary logistic regressions for each combination of power × state affect (**Table 6**). Results showed a marginal effect on engaging for dispositionally happy participants who felt sad and had more bargaining power, OR = 5.45, *p* = 0.07, 95% CI (0.90, 33.14). Thus, those who generally feel happy and have more bargaining power engage 5.45 more often although they felt sad before the negotiation. For one unit increase in dispositional happiness, their probability to engage a negotiation increases from 91.68 to 98.35%.

##### Make a request

The results of the binary logistic regression (**Table 5**) revealed a positive main effect for power, OR = 2.49, *p* < 0.01, 95% CI (1.27, 4.88), and a negative for dispositional sadness, OR = 0.65, *p* = 0.04, 95% CI (0.43, 0.97). Such results mean that powerful participants are 2.49 more likely to make a suboptimal request, while sad participants are 1.54 times less likely to make a similar request. Again, state affect was found to be insignificant to requesting.

Next, we conducted separate logistic regressions for each combination of state affect and bargaining power (**Table 7**). As with engaging, the results revealed a positive effect of perceived happiness on requesting when bargaining power is high and when participants were induced with sadness, OR = 7.97, *p* = 0.01, 95% CI (1.57, 40.51). Put differently, happy participants who are predisposed into sadness, but have bargaining power are 7.97 more likely to initiate a request. Specifically, a unit increase in dispositional happiness increases the probability to make a suboptimal request from 43.05 to 85.81%.

##### Optimize a request

**Table 5** shows that the effects power and perceived sadness have a similar pattern on the decision to optimize a request. The OR value for power was 3.58, *p* < 0.01, 95% CI (1.54, 8.32), which means that powerful participants are 3.58 more likely to optimize. Also, sad individuals are 1.72 less likely to optimize, OR = 0.58, *p* = 0.04, 95% CI (0.35, 0.97). As with the previous stages of the initiation process, the main effect of state affect was immaterial.

As in the previous initiation stages, we conducted four logistic regressions for each cell of the power × state affect condition (**Table 8**). Results indicate a negative, but marginally significant, effect of dispositional sadness when bargaining power is low and participants were induced with sadness, OR = 0.25, *p* = 0.08, 95% CI (0.05, 1.16), or were four times less likely to optimize. Furthermore, we found a positive effect for dispositional happiness when bargaining power is high and participants were induced with sadness, OR = 5.38, *p* = 0.02, 95% CI (1.29, 22.44), making them 5.38 times more likely to optimize a request. Practically, a unit increase in dispositional sadness decreases the probability of optimizing a request from 10.34 to 2.77% when primed into sadness and have low bargaining power. In addition, a unit increase in dispositional happiness makes optimizing more probable (from 20.42 to 57.93%) for those who incidentally feel sad but have more bargaining power.

#### Discussion

The purpose of the second study was twofold. The first was to test whether two incidental emotions (happiness and sadness) have the potency to carry over their effects on negotiation initiation at high vs. low bargaining power conditions. Contrary to our expectations, our findings indicate that induced happiness or sadness had no impact on negotiation initiation, controlling for the effects of trait affect. Hence, happiness and sadness as incidental emotions are no worthy substitutes of bargaining power.

The second, which may explain why state affect had an immaterial impact on negotiation initiation, was to assess the role of trait affect while experiencing different incidental emotions (dual emotion condition) under bargaining power asymmetry. Here, contrary to our expectations, our findings indicate that incidental sadness can increase the likelihood of engaging, requesting and optimizing when coupled with a power advantage and a happy disposition. In addition, we found that when power is high, being ephemerally sad may decrease the likelihood of making a request, but only when dispositional happiness is also low. When happiness is high, the negative effect of sadness is lost. Furthermore, we found that the likelihood of powerless individuals infused with sadness to optimize their request further decreases when they are usually sad. Hence, although increased levels of bargaining power result in a higher initiation, the multiplication of state and dispositional affect plays a critical role in their decision to initiate negotiations.

## General Discussion

In models of negotiation initiation, traits and situational factors are considered as significant driving forces (e.g., Magee et al., 2007; Kong et al., 2011; Kapoutsis et al., 2013; Volkema et al., 2013; Reif and Brodbeck, 2017). In this paper, we posed the question of whether or not working adults facing a negotiation situation under power asymmetry would be willing to initiate (engage a prospective counterpart, make a suboptimal request, and optimize it) depending on their affect (trait and state: happiness and sadness). Specifically, we aimed our attention on two primary discrete emotions (happiness and sadness) which differ in several characteristics that lead to different appraisal tendencies (Smith and Ellsworth, 1985). Collectively, the results of the two studies revealed some interesting patterns about the role of trait affect and its interaction with state affect on negotiation initiation under power asymmetry.

In Study 1, we found that powerful individuals are more likely to engage, request, and optimize, compared to powerless ones. This finding is aligned to the tenets of the Approach-Inhibition Model of power (Keltner et al., 2003) and adds to the mounting evidence (e.g., Magee et al., 2007; Kapoutsis et al., 2013) that power asymmetry is a decisive driving force of negotiation initiation. Also, we drew from the ATF (Lerner and Keltner, 2000, 2001) and argued that happiness and sadness represent two affect dispositions with different appraisal tendencies that each may influence the decision to initiate negotiation under high and low bargaining power conditions. The results showed that trait affect interacts with bargaining power. For all three stages of the initiation process, dispositional sadness reduced the likelihood of initiating although having a relative power advantage. Still, we found no enhancing effect for sadness on initiation, under a relative power disadvantage. This finding is in line with those from other studies which argued that sadness may indeed increase pessimism about future events (Keltner et al., 1993; Smith and Lazarus, 1993; Helweg-Larsen and Shepperd, 2001), increase propensity for risk avoidance (Lerner et al., 2015) and hence neutralize the benefits that stem from processing more relative power. This may also be attributed to the fact that negatively valenced affect constrains individuals from engaging in negotiations because of the fear in reviving a past failure that may enlarge their potential loss (Morris and Keltner, 2000). On the other hand, we found that people who tend to be happy are more likely to make a request even when power is low. Moreover, happy participants were twice as likely to optimize their request, although the probability was statistically insignificant. The finding that they preferred to skip engaging the counterpart may be explained by the fact that happy individuals tend to take more risks while they tend to reside in heuristic processing which requires minimal cognitive effort (Lerner et al., 2015). Meanwhile, trait happiness had no effect on the propensity of powerful individuals to initiate. Such finding adds up to those from other researches that suggest that happy people may overlook situational cues to avoid cognitive effort that could threaten their ability to maintain their pleasant state (Isen, 1984; Wood et al., 1990). Therefore, our results contradict Overbeck et al.’s. (2010) assertion that trait affect is not an influential factor of judgment in powerless situations.

Study 2 was interested in the interaction between trait and state (incidental) affect. Results reveal that the incidental emotions of happiness or sadness per se do not have any impact on negotiation initiation, controlling for bargaining power. However, they interact with trait affect. In line with what we found in Study 1, for those who lack bargaining power, the results show that initiation is less probable no matter their current state of happiness or sadness or their general tendency to be happy or sad. The only exception was observed for optimizing; those who were induced with incidental sadness and were usually sad (congruent trait-state affect) had very little likelihood of optimizing. With this exception, the emotional augmentation of powerless individuals in dual emotion situations received only limited support.

In theory, we also expected that incongruent trait-state affect would influence decision making under relative bargaining power asymmetry. Contrary to the nature of our prediction we found that individuals who tend to be happy is almost sure that they engage a prospective counterpart, but only when there are induced with sadness rather than happiness. This pattern was repeated for requesting and optimizing as well. However, for powerful individuals who tend to be sad, being infused with incidental happiness increased the likelihood to initiate negotiations (engage, request, optimize), but not to a statistically significant degree. This pattern may explain the lack of support to Study’s 1 proposition that powerful, happy individuals would be more likely to initiate negotiations. As shown in Study 2, this may be attributed to state affect. But, instead of an emotional blunting due to emotional incongruence, we found an emotional augmentation. This finding highlights the fascinating interplay between state and trait affect and contributes to the literature on emotional augmentation and blunting (Winterich et al., 2010; Pe and Kuppens, 2012). Although research on this area is still insufficient, our results indicate that powerful individuals with a tendency to be happy need a sad external stimulus to increase the likelihood of initiation; otherwise the positive carry over of trait happiness on initiation becomes inactive. Nonetheless, this pattern was not observed for powerless, sad individuals infused with incidental happiness.

Study 2 findings corroborate George’s (2011) assertion that both positive and negative affect can be useful. For example, Forgas (2007) found that negative mood may promote a more attentive, accommodating and concrete information processing style. In his study, he found that those primed in a negative mood developed more persuasive arguments than those in a positive mood. In another study, Tamir and Ford (2009) suggested that some individuals may be willing to experience negative affect as it may prove useful to attain instrumental gain. Additionally, Kawakami et al. (2014) found that listening to sad music can be perceived as sad but can evoke positive emotional responses. They suggest that if a negative emotion does not constitute any real threat to the individual, then it generates a positive reaction. In our case, people were primed into sadness and happiness using incidents that were unrelated to the context of the negotiation. Thus, although feeling sad, it may have activated the action tendency of happiness and evoked a positive response. Furthermore, Forgas (2013) noted that negative mood (e.g., sadness) relates to less artificial self-handicaps (i.e., avoidance of effort to protect self-esteem from potential failure). Sadness has also been associated with reduced fundamental attribution error. Thus, sad people may be less prone to ignore situational factors (Forgas, 1998). Moreover, Schwarz (1990) noted that those infused with negative mood process information more systematically and carefully while happy individuals preserve their good mood by avoiding cognitive effort and relying on heuristic processing (Clark and Isen, 1982). Finally, because sadness was incidental, our findings may suggest that a happy disposition could motivate powerful individuals to become more attentive to the negotiation opportunity rather than thinking of the unpleasant incident that caused negative mood. As such, combining happiness as a tendency with ephemeral sadness indicates that activating negative emotions (e.g., anger) may increase the likelihood of initiating. Therefore, future research could test whether emotions that combine unpleasantness with activation (e.g., angry) would make individuals more likely to initiate a negotiation.

Overall, although the self-motivating role of positive affect on individuals’ behavior is well documented (George, 2011; George and Dane, 2016), this research shows that happiness is a driving force, but only for those who are powerful and in a sad state. However, we also found that some negative dispositions (i.e., sadness) have the potency to make individuals direct their attention and behavior toward minimizing potential losses from avoiding to negotiate when they find themselves in a powerful position. The interaction of affect × power can also be viewed using power as a reference point. Although we know that power dramatically increases the likelihood of initiating negotiations (Keltner et al., 2003; Magee et al., 2007; Kapoutsis et al., 2013; Volkema et al., 2013), our results indicate that specific affect dispositions may moderate this likelihood.

What this research practically tells negotiators is that to increase the chances of acting upon negotiable opportunities, they need to account for the situational parameters of the prospective negotiation. For example, negotiators may try to enhance relative bargaining power because it facilitates not only engaging but also requesting and optimizing. To increase relative bargaining power, individuals may look for other alternatives, possess resources that are valuable to the other party, impose time constraints on the other party, setting goals and forming if-then plans, etc. (Overbeck and Kim, 2013; Jäger et al., 2017). They need to be aware, however, that such advantage may attenuate for those with a sad disposition. To put it differently, having a happy disposition while in a power disadvantage may not vest individuals from withdrawing or from making and optimizing a request. But, having a sad disposition may block the driving force produced by power advantage. Yet, powerful negotiators with a happy disposition may find it useful to think of sad or negative vicarious experiences (unrelated to the negotiation task) or simply associate with other people who have a sad disposition in life. Such connections may make happy people to become more attentive and give the necessary push to engage in negotiation, making a request, and optimizing it. Therefore, if we view initiation behavior not only as the first step before starting negotiations but also as a critical step throughout the negotiation process (e.g., making requests as the negotiation unfolds), then being able to infuse specific emotions and regulate bargaining power may increase concessions and facilitate cooperative agreements.

As with all vignette studies, the application of these findings comes with several caveats. First, the two studies measured implementation intentions rather than actual behaviors. However, intentions are likely to predict behaviors, particularly when individuals have control over behavior, social reactions are unlikely, and habitual strength is low (e.g., avoiding behavior as a habit) (Webb and Sheeran, 2006). Second, this article focused on two primary discrete emotions, happiness and sadness. These two emotions represent two different semantic components of emotion appraisal models. It would be worthwhile to design similar studies and focus on other emotions that differ in their characteristics (e.g., anger and calmness). Finally, state affect in this study was manipulated using incidental emotions, which were unrelated to that situation. Future research may as well find a different pattern in the results if these emotions arose from a decision at hand (integral emotion; Lerner et al., 2015; Hillebrandt and Barclay, 2017).

Naturally, individuals are more likely to engage in negotiations when they feel that the other party will accommodate their request. Emotions convey information about social interactions and how people perceive the other party (Frijda, 1986), which in turn influences the behavioral response of the recipient (Barry et al., 2004; Van Kleef et al., 2004). Hence, emotions can be contagious and be transmitted from the initiator to the recipient subconsciously (Levy and Nail, 1993). And being able to transfer the positive affect to the other party can influence the judgment that relates to first impressions (Forgas, 2011). To this end, individuals who can prime or regulate their affect and match the situation can control the information sent to counterparts and increase the likelihood of eliciting the desired response. Therefore, future research on negotiation initiation may examine how this pre-negotiation decision making stage is affected by the emotional state of the prospective counterpart, how affect between the two interact, and how emotion regulation may help elicit the desired behavior and response.

Future researchers may also investigate the role of emotions and power not only on the initial decision to negotiate but also when this first attempt has failed (e.g., failing to receive the intended preliminary response). Will the asking party abandon the re-initiation attempt when bargaining power is low, irrespective of his or her affective state or will it similarly influence this second decision? We suggest that even if negotiators have more power initially, re-engaging will be less likely unless coupled with positive affect; otherwise bargaining power will remain a valuable but inactive resource. Antithetically, the party with the lower bargaining power can ignore the initial approach (a tactic that restores power imbalance). If this attempt fails, then inducing negative emotions to the initiator may set the initiation threshold at a higher level and make the asking party reluctant to re-engage.

## Conclusion

We argue that the theoretical and empirical research in negotiations will advance as researchers start looking at early negotiation stages. While demographic and personality factors have heretofore been the primary focus, situational factors may offer greater predictability (Bazerman et al., 2000). Based on these two studies, affect, power, and their interaction appear to hold both theoretical and practical promise for understanding the engaging, requesting, and optimizing phases of the initiation process.

## Author Contributions

IK substantially contributed the conception and design of the two studies, the collection and analysis of the data, and to the writing of the paper. RV contributed to the conception and design of the two studies and made contributions to the writing of the article. AL made substantial contributions to the analysis and interpretation of the data, and to the writing of the paper. All authors approved the current version of the manuscript.

## Conflict of Interest Statement

The authors declare that the research was conducted in the absence of any commercial or financial relationships that could be construed as a potential conflict of interest.

## APPENDIX

### Appendix. Salary Negotiations Scenarios Used In Studies 1 And 2

#### High Bargaining Power

Imagine that you are negotiating with a small but growing company about the terms of their job offer to you. The human resources department has informed you of your likely salary, based on what the previous person in this position earned. The salary might be acceptable to you, but it is considerably lower than what you know similar people make in similar positions (15% less). Although *this job is not your top choice*, it seems promising, but you were expecting a much higher salary. If you want to negotiate any of the terms of the offer, the human resources representative indicates that you must contact the company’s vice president of administration by tomorrow. Although you do not know for sure, *it seems unlikely that the company is considering other applicants for this position*. Also, besides this offer, *you expect other offers from other companies*.

#### Low Bargaining Power

Imagine that you are negotiating with a small but growing company about the terms of their job offer to you. The human resources department has informed you of your likely salary, based on what the previous person in this position earned. The salary might be acceptable to you, but it is considerably lower than what you know similar people make in similar positions (15% less). *The job is your top choice*, but you were expecting a much higher salary. If you want to negotiate any of the terms of the offer, the human resources representative indicates that you must contact the company’s vice president of administration by tomorrow. Although you do not know for sure, it seems likely that *the company is considering other applicants for this position*. Also, *you do not have any other offer that you could rely on*.

Instructions: Looking yourself from a distance and being as realistic as possible, which of the following option would you more likely choose? (appeared in randomized order)

You tell the human resources representative that you will accept/reject the offer, and you do not contact the vice president of administration.

You contact the vice president of administration to talk about the position, hoping that he or she will make a better offer without you requesting a higher salary.

You contact the vice president of administration and request a higher salary than what the human resources department mentioned, but lower than what the previous person in this position earned.

You contact the vice president of administration to request a salary that is at least 15% higher than what the human resources department mentioned.
